# Longitudinal increase of humoral responses after four SARS-CoV-2 vaccinations and infection in MS patients on fingolimod

**DOI:** 10.1177/13524585231207761

**Published:** 2023-11-09

**Authors:** Zoé LE van Kempen, Koos PJ van Dam, Jim BD Keijser, Eileen W Stalman, Laura YL Kummer, Eva MM Strijbis, Maurice Steenhuis, Anja ten Brinke, S. Marieke van Ham, Taco Kuijpers, Theo Rispens, Filip Eftimov, Luuk Wieske, Joep Killestein

**Affiliations:** Department of Neurology, Amsterdam University Medical Centers, Vrije Universiteit, Amsterdam, The Netherlands; Department of Neurology and Neurophysiology, Amsterdam Neuroscience, Amsterdam University Medical Centers, Location AMC, University of Amsterdam, Amsterdam, The Netherlands; Department of Immunopathology, Sanquin Research and Landsteiner Laboratory, Amsterdam University Medical Centers, Amsterdam, The Netherlands; Department of Neurology and Neurophysiology, Amsterdam Neuroscience, Amsterdam University Medical Centers, Location AMC, University of Amsterdam, Amsterdam, The Netherlands; Department of Neurology and Neurophysiology, Amsterdam Neuroscience, Amsterdam University Medical Centers, Location AMC, University of Amsterdam, Amsterdam, The Netherlands; Department of Immunopathology, Sanquin Research and Landsteiner Laboratory, Amsterdam University Medical Centers, Amsterdam, The Netherlands; Department of Neurology, Amsterdam University Medical Centers, Vrije Universiteit, Amsterdam, The Netherlands; Department of Immunopathology, Sanquin Research and Landsteiner Laboratory, Amsterdam University Medical Centers, Amsterdam, The Netherlands; Biologics Laboratory, Sanquin Diagnostic Services, Amsterdam, The Netherlands; Department of Immunopathology, Sanquin Research and Landsteiner Laboratory, Amsterdam University Medical Centers, Amsterdam, The Netherlands; Department of Immunopathology, Sanquin Research and Landsteiner Laboratory, Amsterdam University Medical Centers, Amsterdam, The Netherlands; Swammerdam Institute for Life Sciences, University of Amsterdam, Amsterdam, The Netherlands; Department of Pediatric Immunology, Rheumatology and Infectious Disease, Amsterdam University Medical Centers, Location AMC, University of Amsterdam, Amsterdam, The Netherlands; Department of Immunopathology, Sanquin Research and Landsteiner Laboratory, Amsterdam University Medical Centers, Amsterdam, The Netherlands; Department of Neurology and Neurophysiology, Amsterdam Neuroscience, Amsterdam University Medical Centers, Location AMC, University of Amsterdam, Amsterdam, The Netherlands; Department of Neurology and Neurophysiology, Amsterdam Neuroscience, Amsterdam University Medical Centers, Location AMC, University of Amsterdam, Amsterdam, The Netherlands; Department of Clinical Neurophysiology, St. Antonius Hospital, Nieuwegein, The Netherlands; Department of Neurology, Amsterdam University Medical Centers, Vrije Universiteit, Amsterdam, The Netherlands

**Keywords:** S1P modulators, COVID-19, immunization, antibodies, omicron

## Abstract

**Background:**

Humoral responses after SARS-CoV-2 vaccination are greatly impaired in multiple sclerosis (MS) patients on fingolimod. Effects of repeated vaccination and infections on long-term responses are unclear.

**Methods:**

Prospective study in 60 MS patients on fingolimod measuring humoral responses after up to four vaccinations and 8 months after fourth vaccination.

**Results:**

Anti-WH1 antibody titers increased with each additional vaccination. At long-term follow-up titers increased further and most patients developed new humoral responses against the BA.1 omicron variant.

**Conclusion:**

Repeated SARS-CoV-2 vaccinations boost humoral immunity and, probably together with SARS-CoV-2 infections, induce humoral responses on the long-term in almost all patients.

## Introduction

In patients with multiple sclerosis (MS), fingolimod is associated with severely impaired humoral responses following SARS-CoV-2 vaccination for primary immunization, although risks for (severe) breakthrough infections appear limited.^1–3^ Based on this decreased response, MS patients on fingolimod are offered additional SARS-CoV-2 vaccinations in many countries, albeit with little effect on the humoral immune response after a third vaccination and no effect after a fourth vaccination.^1,2^ However, the number of patients who have experienced a SARS-CoV-2 infection has increased considerably over time, providing an additional boost in immune responses. The combined effect of repeated vaccination and infections on long-term humoral responses in patients on fingolimod is unknown.

In this study, we longitudinally assessed humoral responses after a maximum of four SARS-CoV-2 vaccinations and at long-term follow-up in MS patients on fingolimod.

## Methods

This is a substudy of an ongoing prospective observational multicenter cohort study on immunity against SARS-CoV-2 in various immune-mediated inflammatory diseases (Target-to-B! (T2B); Trial NL8900; Dutch Trial register). The medical ethical committee of the Amsterdam UMC, location AMC (2020.194) approved the study and participants provided written informed consent.

In this study, we included patients with MS, who received at least one SARS-CoV-2 mRNA vaccine, used fingolimod during primary immunization, and with at least one serum sample after any vaccination available for SARS-CoV-2 antibody measurement. Clinical data were retrieved from the medical files. Data on antigen test or polymerase chain reaction proven SARS-CoV-2 infections and vaccinations were collected by digital questionnaires. Samples were obtained by self-performed finger prick at home or by venipuncture in the hospital at 28 days after first, second, third, and booster vaccination. This third vaccination was offered to patients on fingolimod as an additional early booster to complement primary immunization ahead of the regular booster vaccination. As additional vaccinations, participants were vaccinated with mRNA vaccines. Approximately 8 months after booster vaccination, from September to November 2022, a long-term follow-up sample was obtained in a subgroup of participants who donated blood through venipuncture during primary immunization. Healthy controls were included when a long-term follow-up sample and at least one sample after vaccination were available.

SARS-CoV-2 antibodies against Wuhan-Hu-1 (WH1) and Omicron BA.1 receptor-binding domain (RBD) were measured at Sanquin laboratory using an IgG-specific quantitative enzyme-linked immunosorbent assay (ELISA).^4^ The cut-off for a positive ELISA was 4.0 arbitrary units per ml (AU/mL), representing 99% specificity in pre-outbreak samples.^4,5^

We assessed the median antibody titer and proportion of seropositive participants at each time point, and in patients with paired samples at sequential vaccinations, we compared changes in titers and seropositivity from second to third vaccination, from third to booster, and from booster to long-term follow-up using the Wilcoxon signed-rank test and McNemar’s test, respectively. The number of patients with SARS-CoV-2 infections was too low to allow comparison between patients with or without infection. Data analysis was performed in R version 4.3.0 (R foundation for Statistical Computing, Vienna, Austria).

## Results

Sixty patients and 43 healthy controls were included in this study (Supplemental Figure 1). Participant characteristics are summarized in Table 1. Three patients switched to other disease modifying therapies (DMT) after primary immunization (Supplemental Table 1). Not all samples were available for all time points (Supplemental Table 3). Three patients experienced a SARS-CoV-2 infection before primary immunization. At successive sampling time points, the cumulative proportion of samples from patients with a previous SARS-CoV-2 infection increased from 1/44 (2.3%) after first vaccination, 2/52 (3.8%) after second vaccination, 5/55 (9.1%) after third vaccination, 6/33 (18.2%) after booster vaccination, and 11/15 (73.3%) at long-term follow-up.

**Table 1.** Participant characteristics.

	MS patients on fingolimod (*N* = 60)	Healthy controls (*N* = 43)
*Age*		
Years, mean (SD)	44.2 (9.1)	51.3 (7.8)
*Sex*		
Female	38 (63.3)	26 (60.5)
Male	22 (36.7)	17 (39.5)
*Number of vaccines in primary immunization*		
One	2 (3.3)	1 (2.3)
Two	58 (96.7)	42 (97.7)
*Vaccine received in primary immunization*		
BNT162b2 (Pfizer-BioNTech)	31 (51.7)	0 (0)
CX-024414 (Moderna)	29 (48.3)	43 (100)
Additional vaccinations*		
Third and booster	39 (65.0)	0 (0)
Third only	17 (28.3)	0 (0)
Booster only	1 (1.7)	42 (97.7)
None	3 (5.0)	1 (2.3)
*Time between vaccinations in days, median [IQR]*		
First to second	35 [35–36]	42 [42–42]
First to third	138 [132–160]	–
First to booster	238 [228–245]	239 [232–247]
First to long-term FU sample	482 [473–508]	506 [495–518]

FU: follow-up; IQR: interquartile range; MS: multiple sclerosis; SD: standard deviation.

* Additional vaccinations were either BNT162b2 (Pfizer-BioNTech) or CX-024414 (Moderna) independent of which vaccine was received in primary immunization, an early booster (third vaccination) was offered to patients on fingolimod before the regular booster.

**Figure 1.**
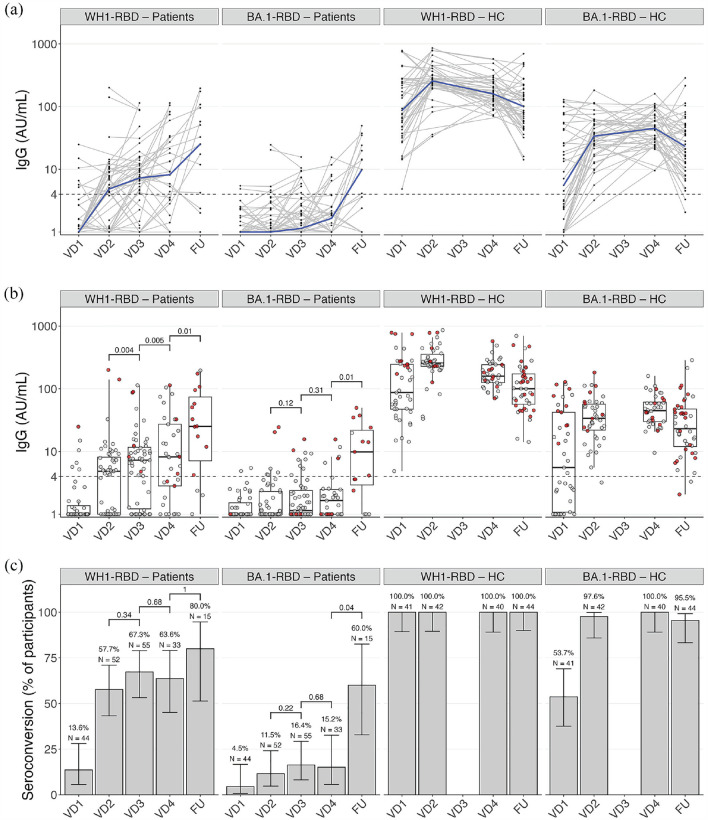
Humoral responses after repeated SARS-CoV-2 vaccination, showing humoral responses 28 days after first (VD1), second (VD2), third (VD3), and booster (VD4) SARS-CoV-2 vaccination, and at long-term follow-up (FU) for antibodies against the Wuhan-Hu-1 (WH1) and Omicron BA.1 RBD in patients on fingolimod and healthy controls. Antibody titers over time after repeated vaccinations, with the dotted line showing threshold for seropositivity, individual trajectories (gray) and median (blue) (a), and measurements with (red dots) and without (gray dots) previous SARS-CoV-2 infection (b). (c) Proportions of seropositivity. AU: arbitrary units; HC: healthy controls; RBD: receptor-binding domain; VD: vaccine dose; WH1: Wuhan-Hu-1.

Humoral responses are shown in Figure 1(a)–(c). In patients, the WH1-RBD titer increased significantly from second to third and from third to booster vaccination (*p* = 0.004 and *p* = 0.005, respectively). Seropositivity was observed in 6/44 (13.6%, 95% CI 5.7–28.0) after first vaccination, 30/52 (57.7%, 95% CI 43.3–71.0) after second, 37/55 (67.3%, 95% CI 53.2–79.0) after third, and 21/33 (63.6%, 95% CI 45.1–79.0) after booster vaccination. Proportions of seropositivity in paired samples at second and third, and at third and booster vaccination did not differ (*p* = 0.34 and *p* = 0.68, respectively). BA.1-RBD humoral response remained low until after booster vaccination and seropositivity was observed in 5/33 (15.2%, 95% CI 5.7–32.7) after booster vaccination. Results of a sensitivity analysis on patients without switch in DMT at booster vaccination are shown in Supplemental Table 2.

A long-term follow-up sample was available in 15 patients. In paired samples, the median WH1-RBD titer at long-term follow-up was higher compared to the titer after booster vaccination (*p* = 0.01), whereas seropositivity did not increase significantly (12/15 (80.0%, 95% CI 51.4–94.7), *p* = 1). The median BA.1-RBD titer at long-term follow-up also increased from booster vaccination to long-term follow-up (*p* = 0.01), as well as seropositivity 9/15 (60.0%, 95% CI 32.9–82.5, *p* = 0.04).

## Discussion

We observed a gradual increase in antibody titers following repeated vaccinations and (breakthrough) infections in MS patients on fingolimod, and approximately 18 months after the start of the pandemic, most patients had a well measurable humoral response. Moreover, we show that the antibody repertoire can broaden over time and that new, in this case anti-BA.1, responses can develop. Together, these findings show that MS patients on fingolimod eventually develop and maintain a humoral response, although it should be noted that antibody titers remain considerably lower compared to titers that have been found in controls or patients on other immunosuppressants.^6^

Other studies on the effect of repeated vaccination in MS patients on fingolimod differed in findings on seropositivity, possibly due to small groups. König et al.^1^ reported seropositivity in 72% (21/29) patients after third vaccination compared to 55% (16/29) after primary immunization. In another cohort, 33% (7/21) of patients were seropositive after primary immunization, and 45% (13/29) were seropositive after third vaccination.^2^ This is lower than the seropositivity proportions in our cohort, which are similar to the findings from König et al. Due to a low number of prior infections, we were unable to separate the individual effects of repeated vaccinations and infections. Arguably, this is less important as the prevalence of SARS-CoV-2 infections has increased considerably over time. At long-term follow-up, antibody titers remained high, either from the booster vaccination, infections, or a combination of both. To our knowledge, we are the first to report on the humoral response after up to four SARS-CoV-2 vaccinations and at long-term follow-up, in a fairly large group of patients on fingolimod.

Strengths of this study are the longitudinal follow-up, measurement of two different types of antibody responses and a relatively large cohort. Limitations are the combination of vaccinations and intercurrent infections making the observed humoral response less generalizable, only mRNA vaccines as additional vaccinations, the absence of cellular immunity measurements and monitoring subclinical infections, and only a small group at long-term follow-up making findings on this timepoint less generalizable.

In conclusion, in our study, almost all MS patients on fingolimod develop an adapting humoral response due to repeated vaccinations and infections at long-term follow-up.

## Supplemental Material

sj-docx-1-msj-10.1177_13524585231207761 – Supplemental material for Longitudinal increase of humoral responses after four SARS-CoV-2 vaccinations and infection in MS patients on fingolimod Supplemental material, sj-docx-1-msj-10.1177_13524585231207761 for Longitudinal increase of humoral responses after four SARS-CoV-2 vaccinations and infection in MS patients on fingolimod by Zoé LE van Kempen, Koos PJ van Dam, Jim BD Keijser, Eileen W Stalman, Laura YL Kummer, Eva MM Strijbis, Maurice Steenhuis, Anja ten Brinke, Marieke SM van Ham, Taco Kuijpers, Theo Rispens, Filip Eftimov, Luuk Wieske and Joep Killestein in Multiple Sclerosis Journal
