# Supplementary material for: Longitudinal increase of humoral responses after four SARS-CoV-2 vaccinations and infection in MS patients on fingolimod
Source: Mult Scler. 2023 Nov 9;30(3):443–7. doi: 10.1177/13524585231207761 (PMC10935618; doi:10.1177/13524585231207761)
Supplement: sj-docx-1-msj-10.1177_13524585231207761 – Supplemental material for Longitudinal increase of humoral responses after four SARS-CoV-2 vaccinations and infection in MS patients on fingolimod [file sj-docx-1-msj-10.1177_13524585231207761.docx]

**Supplementary content**

*Figure 1: Study flowchart*

DMT: disease modifying therapy; MS: multiple sclerosis

*Table 1. Disease modifying therapies during vaccinations*

|  | N (%) |
| --- | --- |
| DMT during primary immunisation |  |
| Fingolimod | 60 (100) |
| DMT at time of third vaccination |  |
| Fingolimod | 56 (100) |
| DMT at time of booster vaccination |  |
| Fingolimod | 37 (92.5) |
| Anti-CD20 | 2 (5.0) |
| Dimethylfumarate | 1 (2.5) |

*No vaccination registered

DMT: disease modifying therapy

*Table 2. Sensitivity analysis*

Results from a sensitivity analysis on the humoral response after booster vaccination. For this analysis, we excluded three patients that were not on fingolimod anymore during the booster vaccination and repeated the original analysis.

|  | VD3 | VD4 | Long-term FU | Third to booster difference | Booster to long-term FU difference |
| --- | --- | --- | --- | --- | --- |
| *Anti-WH1-RBD* |  |  |  |  |  |
| Seroconversion,  n/N (%) | 36/52  (69.2) | 19/30  (63.3) | 11/13 (84.6) | p=0.37 | p=1 |
| Antibody titer,  Median (IQR) | 7.3  (1.3–12.1) | 8.2  (2.9–25.8) | 32.7  (11.9–95.3) | p=0.012 | p=0.02 |
| *Anti-BA.1-RBD* |  |  |  |  |  |
| Seroconversion,  n/N (%) | 9/52  (17.3) | 4/30  (13.3) | 8/13  (61.5) | p=1 | p=0.07 |
| Antibody titer,  Median (IQR) | 1.2  (1.0–2.6) | 1.3  (1.0–2.4) | 14.1  (3.6–24.1) | p=0.56 | p=0.02 |

FU: follow-up; IQR: interquartile range; RBD: receptor-binding domain; VD3: third vaccination; VD4: booster vaccination

*Table 3. Overview of samples per timepoint*

|  | VD1 | VD2 | VD3 | VD4 | Long-term FU |
| --- | --- | --- | --- | --- | --- |
| Samples of patients | (N=55) | (N=55) | (N=55) | (N=37) | (N=17) |
| Fingerprick, complete | 44 (80.0) | 52 (94.5) | 55 (100) | 33 (89.2) | 8 (47.1) |
| Fingerprick, not enough volume | 4 (7.3) | 0 (0) | 0 (0) | 0 (0) | 0 (0) |
| Fingerprick, tube not returned | 7 (12.7) | 3 (5.5) | 0 (0) | 4 (10.8) | 2 (11.8) |
| Venipuncture, complete | 0 (0) | 0 (0) | 0 (0) | 0 (0) | 7 (41.2) |
| Samples of healthy controls | (N=42) | (N=41) | (N=0) | (N=41) | (N=43) |
| Fingerprick, complete | 41 (97.6) | 41 (100) | - | 39 (95.1) | 32 (74.4) |
| Fingerprick, not enough volume | 1 (2.4) | 0 (0) | - | 0 (0) | 0 (0) |
| Fingerprick, tube not returned | 0 (0) | 0 (0) | - | 2 (4.9) | 0 (0) |
| Venapuncture, complete | 0 (0) | 0 (0) | - | 0 (0) | 11 (25.6) |

Samples were collected at 28 days after first (VD1), second (VD2), third (VD3), and booster (VD4) vaccination and in a subgroup of patients a long-term follow-up sample was collected after the booster vaccination sample (long-term FU).
